# Modulating Functionalized Poly(ethylene glycol) Diacrylate Hydrogel Mechanical Properties through Competitive Crosslinking Mechanics for Soft Tissue Applications

**DOI:** 10.3390/polym12123000

**Published:** 2020-12-16

**Authors:** Rachel Chapla, Mera Alhaj Abed, Jennifer West

**Affiliations:** Department of Biomedical Engineering, Duke University, Durham, NC 27708, USA; rachel.chapla@duke.edu (R.C.); alhajabed.mera@gmail.com (M.A.A.)

**Keywords:** hydrogel, tissue engineering, cell culture scaffold, substrate stiffness, mechanotransduction, PEGDA

## Abstract

Local mechanical stiffness influences cell behavior, and thus cell culture scaffolds should approximate the stiffness of the tissue type from which the cells are derived. In synthetic hydrogels, this has been difficult to achieve for very soft tissues such as neural. This work presents a method for reducing the stiffness of mechanically and biochemically tunable synthetic poly(ethylene glycol) diacrylate hydrogels to within the soft tissue stiffness regime by altering the organization of the crosslinking sites. A soluble allyl-presenting monomer, which has a higher propensity for chain termination than acrylate monomers, was introduced into the PEG-diacrylate hydrogel precursor solution before crosslinking, resulting in acrylate-allyl competition and a reduction in gel compressive modulus from 5.1 ± 0.48 kPa to 0.32 ± 0.09 kPa. Both allyl monomer concentration and chemical structure were shown to influence the effectiveness of competition and change in stiffness. Fibroblast cells demonstrated a 37% reduction in average cell spread area on the softest hydrogels produced as compared to cells on control hydrogels, while the average percentage of neural cells extending neurites increased by 41% on these hydrogels, demonstrating the potential for this technology to serve as a soft tissue culture system.

## 1. Introduction

Cell behavior and phenotype are largely influenced by interactions with their local environment. It has been well established that cells receive signals from biochemical factors in their environment such as extracellular proteins and adhesive ligands. In more recent years, cells were also shown to sense and respond to the stiffness of their environment [1,2,3,4]. As cells spread and migrate, they generate contractile forces through contact with their surroundings. The stiffness of the local environment determines the magnitude of the tension created, which subsequently influences cell cytoskeletal arrangement and signal pathway activation, and thus downstream cell activity [5,6,7]. Cell viability, adhesion, spreading, and migration are all influenced by matrix stiffness [8,9]. Resultantly, stem cells differentiate down cell lineages corresponding to the tissue type that matches their substrate in stiffness [1]. Thus, the environmental stiffness of tissue culture systems for both investigative and therapeutic applications can greatly impact the activity and fate of cultured cells. It has been thoroughly demonstrated in the literature that matrix stiffness is particularly influential in systems supporting culture of cells from especially soft tissues such as neural cells. Neural cells demonstrate increased neurite extension in softer environments [10,11,12], and neural stem and progenitor cells favor neurogenesis over astrogenesis on most compliant substrates at stiffnesses of 100–1000 Pa, generally optimized around 500 Pa [13,14,15,16]. Even cells with greater pluripotency such as mesenchymal stem cells (MSCs), embryonic stem cells, and induced pluripotent stem cells all favor neuronal differentiation when cultured on substrates ≤1 kPa modulus [1,17,18]. Therefore, successful design of platforms to model soft tissue interactions necessitates a mechanism for controlling modulus within the natural stiffness regime of these tissues (0.1–1 kPa) [19,20].

To best understand cell interactions in order to harness their therapeutic potential, key aspects of the cells’ natural environment must be recapitulated as accurately as possible; thus, in vitro culture systems can be designed to mimic in vivo cell environments. In vivo, cells exist within a fibrous protein extracellular matrix (ECM) that provides adhesive, biochemical, and mechanical cues that significantly influence cell behavior [21]. Thus, there has been a shift in cell culture systems from stiff glass and plastic surfaces to ECM-mimetic compliant platforms. Hydrogels are commonly used because they mimic the soft, hydrated linked network of macromolecules comprising the ECM [22,23,24]. Cell environments, particularly stem cell niches, are highly specified and dynamic, and thus hydrogels must be tailored precisely to model these different tissue conditions.

Many different hydrogel culture platforms exist, fabricated from both naturally-derived and synthetic polymers. Naturally-derived polymers such as collagen, fibrin, and hyaluronan have been frequently implemented in matrices for neural cell culture [25,26,27,28,29] as they are able to form hydrogels that are highly compliant and accurately recapitulate soft tissue stiffness. Natural polymers are also good models of the tissue environment as they are bioactive and cell adhesive without modification. However, this characteristic presents considerable drawbacks as well. While natural polymers present bioactivity, it is with little tunability or control. Furthermore, altering stiffness is achieved by varying polymer concentration, which also influences biomolecule density in these systems. Mechanical and biochemical properties cannot be decoupled to investigate independently [22,30]. Additionally, large batch-to-batch variability can confound reproducibility of studies [22]. Thus, naturally derived polymers do not provide a highly controlled and well-characterized environment for investigating the influences of environmental cues on soft tissue behavior. Alternatively, synthetic polymers provide a material that is highly controlled and customizable but requires modifications and tailoring to model the cells’ natural environment. Poly(acrylamide) [1,16,18,24,31,32] and poly(ethylene glycol) (PEG) [10,26,33] are commonly implemented in platforms for soft tissue cell culture. Synthetic hydrogels allow for repeatable control of material properties.

PEG is a particularly advantageous polymer for modeling a broad range of tissue environments: due to its hydrophilic structure and flexible backbone, PEG resists non-specific protein adsorption, which prevents uncontrolled interactions with cells. This makes PEG a bioinert “blank slate” that can be engineered as desired to impart specific influences on cells. When PEG chain ends are functionalized with acrylate groups to create PEGDA (PEG-diacrylate), free radical-mediated crosslinking at chain ends creates a covalently linked network that forms a hydrogel [34]. Biomolecules conjugated to monoacrylate-PEG can be grafted into the network at desired densities, independent of other material properties. Hydrogel mechanical modulus is instead tuned by altering the PEG molecular weight or polymer density. This decoupled control over biochemical and mechanical hydrogel properties allows for independent investigation of the influence of each of these parameters on cell behavior [30]. Due to the customizability of this system, PEG-based hydrogels have been designed to model many different tissue environments. Functionalized PEGDA hydrogels have formed the foundation for investigation of a range of cell behaviors such as the role of macrophages in angiogenesis, tumor cell interactions, and the influence of different adhesive ligands on vasculature formation [35,36,37]. While current methods for manipulating PEG hydrogel stiffness have proven appropriate for many tissue applications, they are limited in applicability for soft tissue modeling. Hydrogels are unable to form below a minimum polymer density due to the critical micelle concentration cutoff. At this minimum density, PEGDA hydrogels still have a modulus of greater than 1 kPa; thus, PEGDA can rarely form stable hydrogels within a neural stiffness range [30]. This indicates the need for a mechanism independent of polymer density for increasing PEG-based hydrogel compliance to that of the soft tissue regime for neural tissue engineering applications.

We have previously demonstrated a novel method for decreasing PEGDA hydrogel compressive stiffness to within the neural regime by incorporating a short side chain known as allyloxycarbonyl, or alloc, for short, onto a site on the polymer backbone. The allyl from this group participates in the addition polymerization of vinyl groups at crosslinking sites with lower free-radical propagation than acrylate. Competition between acrylate chain endgroups and alloc during crosslinking results in a decreased hydrogel compressive modulus without preventing gelation or altering the hydrogel diffusive properties or biochemical signal density [30]. However, the level of control over mechanical properties for this system still leaves room for improvement. As the alloc group in this system is a sidegroup on a degradable peptide conjugated into the PEGDA backbone, the molar ratio of acrylate to alloc cannot be adjusted to precisely tune stiffness. Furthermore, this technology is not easily applied to other acrylate-functionalized materials, as it requires specialized synthesis for alloc sidegroup presentation. It would be beneficial to harness the competitive mechanism of alloc for reducing hydrogel stiffness with more facile tunability over a range of stiffnesses and without individual polymer syntheses needed for each condition. Here, we developed a system to exert greater control over PEGDA hydrogel mechanical properties by soluble implementation of alloc-presenting monomers into the polymer mix before gelation. By altering the allyl-to-acrylate molar ratio, we can effectively control PEGDA hydrogel stiffness in a dose-dependent manner down to ~300 Pa compressive modulus without influencing gelation or biochemical density. Furthermore, we show that this behavior is not limited to alloc alone, and that other allyl-presenting monomers can be implemented as well to produce a variety of stiffness conditions, with monomer structure determining effectiveness of allyl competition. This mechanism was shown to be allyl-specific, as acrylate monomers did not reduce hydrogel stiffness, and is suggested to be caused by the difference in propensity between acrylate and allyl groups for free radical propagation [30]. Finally, we show that this material system provides a favorable environment for soft tissue culture, with improved neurite outgrowth on the softest matrices synthesized with a high concentration of soluble alloc.

## 2. Materials and Methods

### 2.1. Polymer Synthesis

Poly(ethylene glycol) diacrylate was synthesized by dissolving 6 kDa poly(ethylene glycol) (PEG, Sigma, St. Louis, MO, USA) in anhydrous dichloromethane (DCM, Fisher Scientific, Waltham, MA, USA) with 2× molar excess of triethylamine (TEA, Sigma, St. Louis, MO, USA). 2× molar excess of acryloyl chloride (Sigma, St. Louis, MO, USA) was added in a dropwise fashion and the mixture was allowed to react overnight under argon. After the reaction, K_2_CO_3_ (Fischer Scientific, Waltham, MA, USA) was added to separate the mixture into phases and release CO_2_. Any remaining aqueous phase was removed by addition of MgSO_4_ (Fisher Scientific, Waltham, MA, USA) and the organic phase, containing PEG, was precipitated with cold diethyl ether (VWR Analytical, Radnor, PA, USA), then filtered and dried under vacuum. Acrylation was verified via proton nuclear magnetic resonance (^1^H-NMR—500 MHz Varian/Agilent VNMRS spectrometer (Agilent, Santa Clara, CA, USA) in chloroform-D (ACROS Organics, Fair Lawn, NJ, USA)).

To promote cell adhesion to the PEG hydrogel matrix, acrylated PEG-conjugated peptides (or PEGylated peptides) were made as follows: adhesive integrin-binding ligand peptide arg-gly-asp-ser (RGDS; GenScript, Piscataway, NJ, USA) was conjugated to 3.2 kDa acrylate-PEG-succinimidyl valerate (acryl-PEG-SVA; Laysan Bio, Arab, AL, USA) by dissolving 1 mol acryl-PEG-SVA with 1.2 mol RGDS (0.2 molar excess) in anhydrous dimethyl sulfoxide (DMSO, Sigma, St. Louis, MO, USA), then adding 2 moles N,N-diisopropylethylamine (DIPEA, Sigma, St. Louis, MO, USA) per 1 mol acryl-PEG-SVA, and allowing to react overnight at room temperature with constant agitation. The product was dialyzed against ultrapure water using a 3.5 kDa MWCO regenerated cellulose membrane (Repligen, Waltham, MA, USA) and then the product was lyophilized. Conjugation efficiency was assessed via gel permeation chromatography (GPC) with an evaporative light scattering detector (Polymer Laboratories, Amherst, MA, USA).

### 2.2. Hydrogel Synthesis

First, glass slides were treated with Sigmacote (Sigma, St. Louis, MO, USA) to impart hydrophobicity and glass coverslips were modified to present methacrylate groups by first etching with Piranha (30% H_2_O_2_ (EMD Millipore, Burlington, MA, USA) + 70% H_2_SO_4_ (VWR Analytical, Radnor, PA, USA)) for 1.5 h, followed by soaking for 48 h in 95% ethanol (Decon Labs, King of Prussia, PA, USA) with 2% *v*/*v* 3-(trimethoxysilyl) propyl methacrylate) (TMSPA, Sigma, St. Louis, MO, USA).

PEGDA was dissolved at desired concentration in 20 mM HEPES-buffered saline (pH 8.3) with 1.5% *v*/*v* triethanolamine (TEOA, Sigma, St. Louis, MO, USA) (pH 8.3), 0.35% *v*/*v* n-vinyl-2-pyrrolidone (NVP, ACROS Organics, Fair Lawn, NJ, USA/Sigma, St. Louis, MO, USA), and 10 µM Eosin Y (Sigma, St. Louis, MO, USA) for light-activated crosslinking.

**Figure 1:**
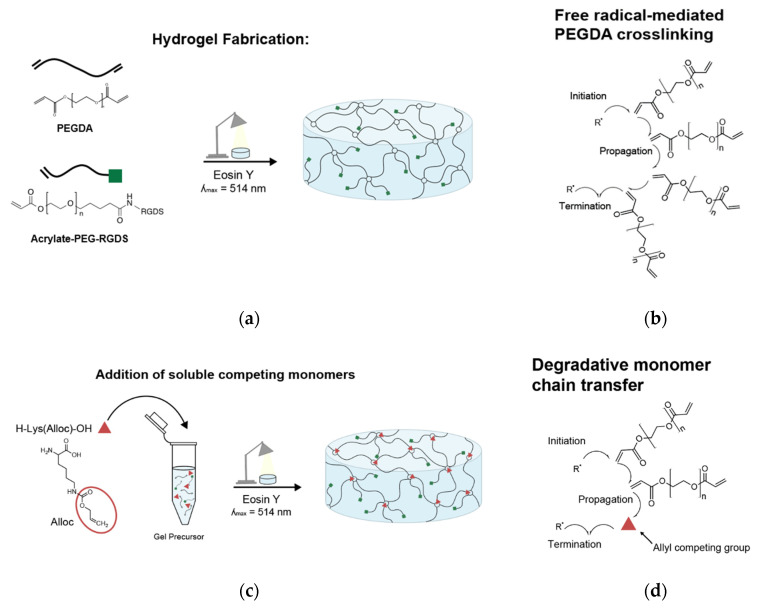
PEGDA hydrogel fabrication and crosslinking schematic (**a**) PEGDA + PEG-RGDS hydrogel fabrication; (**b**) addition polymerization reaction occurring at crosslinking sites in control gels; (**c**) PEGDA + PEG-RGDS + H-Lys(alloc)-OH gel fabrication; (**d**) acrylate—alloc crosslinking competition.

For hydrogels to be seeded with cells, PEG-RGDS was also dissolved in the same photoinitiator solution and combined with the PEGDA solution (Figure 1a). For hydrogels containing competitive vinyl monomers, the monomers were dissolved in the same solution and added to the polymer solutions (Figure 1c). Competitive monomers used were: H-Lys(alloc)-OH, 2-methyl-3-buten-2-ol, methyl-3-butenoate, 3-butenoic acid, and 2-carboxyethyl acrylate (all from Sigma, St. Louis, MO, USA). 2-carboxyethyl acrylate is stored with 900–1100 ppm polymerization inhibitor monomethyl ether hydroquinone (MEHQ). Thus, it was first mixed with anhydrous hexane (Sigma, St. Louis, MO, USA) at 2 g 2-carboxyethyl acrylate per 50 mL hexane and stirred on ice for 2 h. The MEHQ dissolved in hexane while the pure 2-carboxyethyl acrylate remained separated from the n-hexane in a distinct phase and was collected.

To form a hydrogel, this gel precursor solution was pipetted onto a Sigmacote-treated glass slide, between polydimethylsiloxane (PDMS, Electron Microscopy Sciences, Hatfield, PA, USA) spacers. The spacers were 1 mm thick for gels made for mechanical testing and 0.4 mm thick for gels made for cell seeding. Then, a methacrylate-modified coverslip was placed on the spacers, trapping the gel precursor in a cylindrical shape between glass surfaces [35]. The gel precursor solution was then exposed to white light from a Fiber-Lite High Intensity Illuminator Series 180 lamp (Dolan-Jenner Industries, Boxborough, MA, USA; 275 mW/cm^2^) for 50 s (Figure 1a,c).

### 2.3. Mechanical Testing

Gels made 1 mm in height were swollen overnight in phosphate buffered saline (1× PBS, pH 7) at 37 °C, then rinsed with PBS. They were placed on the parallel plates of an RSA III microstrain analyzer (TA Instruments, New Castle, DE, USA) and compressed at a strain rate of 0.003 mm/sec. From the resulting stress–strain curve, the slope of the linear region immediately following the toe region (65–70% strain) was taken as the compressive modulus (Figure S1). For each soluble vinyl monomer, gels were tested with vinyl to acrylate molar ratios of 0:1, 1:1, 2:1, 3:1, and 4:1. Four hydrogels were tested per group.

### 2.4. Biomolecule Density

3.5% *w*/*v* PEGDA hydrogels were made with 3.5 mM PEG-RGDS with and without 10.75 mg/mL H-Lys(alloc)-OH, corresponding to 4:1 allyl:acrylate molar ratio. In addition, 7.5 uM of the 3.5 mM PEG-RGDS was tagged with Alexafluor488 (Thermofisher, Waltham, MA, USA). Hydrogels were allowed to swell in 1× PBS for 3 h, then PBS was exchanged 3×. Images of each hydrogel (*n* = 3 hydrogels per condition) were taken at 480 nm excitation/535 nm emission with a Zeiss Axiovert 135 epifluorescence microscope (Zeiss, Oberkochen, Germany) (5× CP-Achromat objective, NA: 0.12). Three circular regions of interest (ROIs) were randomly selected from each image and were evaluated for average pixel intensity. Background fluorescence was subtracted using the intensity values from a control hydrogel with no PEG-RGDS-A488.

### 2.5. Cell Culture

PC-12 cells (ATCC CRL-1721, ATCC, Manassas, VA, USA) were cultured and expanded in suspension culture in complete growth media (RPMI 1640 ATCC modification, Thermofisher, Waltham, MA, USA) supplemented with 10% *v*/*v* fetal bovine serum (FBS, R&D Systems, Minneapolis, MN, USA), 5% *v*/*v* heat inactivated horse serum (HIHS, Sigma, St. Louis, MO, USA), 100 u/mL penicillin, 0.1 mg/mL strepomycin (P/S, VWR Lifescience, Radnor, PA, USA), and 0.1% *v*/*v* bovine serum albumin (BSA, Sigma, St. Louis, MO, USA). Before seeding on hydrogels, cells were stimulated for 48 h in neural differentiation media (RPMI 1640, ATCC modification (ThermoFisher, Waltham, MA, USA) +1% FBS + 0.5% HIHS + 100 U/mL penicillin, 0.1 mg/mL strepomycin (P/S) + 0.1% BSA + 100 ng/mL nerve growth factor 2.5S (Promega, Madison, WI, USA)). Human umbilical vein endothelial cells (HUVECs, pooled donors, Lonza, Basel, Switzerland) were cultured in Endothelial Cell Growth Medium 2 (Promocell, Heidelberg, Germany) and 100 U/mL penicillin, 0.1 mg/mL streptomycin, 0.92 mg/mL L-glutamine (GPS, Corning, Tewksbury, MA, USA). HUVECs were used at passage 6 for cytotoxicity testing. NIH 3T3 fibroblasts were cultured in Dulbecco’s Modified Eagle’s Medium, High Glucose (Sigma, St. Louis, MO, USA) with 10% *v*/*v* bovine calf serum (BCS, Sigma, St. Louis, MO, USA) and 100 U/mL penicillin, 0.1 mg/mL streptomycin, 0.92 mg/mL L-glutamine (GPS). All cells were maintained at 37 °C with 5% CO_2_.

### 2.6. Cytotoxicity

3.5% *w*/*v* PEGDA hydrogels with 3.5 mM PEG-RGDS with and without 10.75 mg/mL H-Lys(alloc)-OH, corresponding to 4:1 allyl:acrylate, were photocrosslinked and allowed to swell in media overnight at 37 °C. Hydrogels were then rinsed with media, and NIH 3T3 cells and HUVECs were passaged in 0.05% Trypsin + Ethylenediaminetetraacetic acid (EDTA) (Corning, Tewksbury, MA, USA) and seeded on hydrogels at 5000 cells/cm^2^. At 72 h, the cells were tested for viability using a Live/Dead cytotoxicity assay (ThermoFisher, Waltham, MA, USA): cell media was removed and replaced with 1× PBS containing 4 µM ethidium homodimer (EthD-1) and 24 µM calcein AM. The hydrogels were incubated in this solution for 15 min at 37 °C. Then, the cells were imaged with a Ziess Axiovert 135 epifluorescence microscope at 480 nm excitation/535 nm emission for calcein AM and 560 nm excitation/645 nm emission for EthD-1 (5× CP-Achromat objective, NA: 0.12). 4 fields of view were taken of each hydrogel and all live (green) and dead (red) cells were quantified using ImageJ (National Institutes of Health, Bethesda, MD, USA) (>2400 cells per condition). The percentage of live cells was calculated.

### 2.7. Fibroblast Spreading

PEGDA hydrogels with 3.5 mM PEG-RGDS were made as described above. Polymer concentration and alloc competition were varied to manipulate the hydrogel mechanical properties. Hydrogels were made at 10% PEGDA (42 kPa), 3.5% PEGDA (5.1 kPa), and 3.5% PEGDA + 4:1 alloc (10.75 mg/mL H-Lys-alloc-OH, 0.32 kPa) Hydrogels were allowed to swell in media overnight at 37 °C, then rinsed with media, and then NIH 3T3 cells were passaged and seeded on hydrogels at 5000 cells/cm^2^. Phase images of cells were taken at 24 h after seeding at on a Zeiss Axiovert 135 microscope (20× Plan-Neofluar objective, NA: 0.5). Images were analyzed in ImageJ, where each cell was traced and spread area was calculated. At least 280 cells were counted per condition.

### 2.8. Neurite Outgrowth

PEGDA hydrogels with 9.6 mM PEG-RGDS were made as described above. Hydrogels were made at 3.5% PEGDA (5.1 kPa) and 3.5% PEGDA + 4:1 alloc (10.75 mg/mL H-Lys-alloc-OH, 0.32 kPa). Hydrogels were crosslinked and allowed to swell in PC-12 neural differentiation media overnight at 37 °C and rinsed with media. NGF-stimulated PC-12 cells were passaged and seeded on hydrogels at 1000 cells/cm^2^. Neural differentiation media was exchanged every 48 h, and images were taken at day 7. Eleven fields of view were taken from each hydrogel (*n* = 9 hydrogels per condition) on a Zeiss Axiovert 135 using phase contrast (10× Plan-Neofluar objective, NA: 0.3). All single cells (not in contact with other cells) were counted. The total percentage of cells displaying neurites was quantified for each hydrogel of each condition. A cell was defined as neurite (+) if it displayed a neurite greater than or equal to two cell diameters in length. (Neurites were traced using ImageJ software: the freehand line drawing tool + measure function were used to determine the length of the longest neurite from each cell. For more complex branching morphologies, the semi-automatic tracing segmentation plugin Simple Neurite Tracer (National Institutes of Health, Bethesda, MD, USA) was implemented with default settings [38]).

### 2.9. Statistical Analysis

All statistical analyses were performed with JMP Pro 13 software (SAS Institute, Cary, NC, USA). Data sets were analyzed using a student’s *t*-test for comparisons between two groups and analysis of variance (ANOVA) followed by a Tukey’s Honest Significant Difference (HSD) post-hoc test to compare across multiple groups. For all tests, *p*-values less than 0.05 are considered significant. All data values are reported as mean ± standard deviation. Fibroblast spreading distributions were analyzed with pairwise nonparametric Kolmogorov–Smirnov asymptotic tests with a significance threshold of *p* < 0.0167 (Bonferroni-corrected for the three pairwise tests).

## 3. Results

### 3.1. Hydrogel Fabrication

Here, we constructed PEGDA-based gels with stiffness from 5.1 ± 0.48 kPa to 0.32 ± 0.09 kPa by implementing a competitive monomer that interferes with acrylate crosslinking to reduce the stiffness of the overall hydrogel network structure without preventing gel formation. The fabrication process is shown in Figure 1b. Before crosslinking PEGDA gels by exposing them to white light, a soluble H-Lys(alloc)-OH monomer was mixed into the aqueous gel precursor solution at different concentrations corresponding to alloc group to acrylate group molar ratios of 0:1, 1:1, 2:1, 3:1, and 4:1. Stable gels formed for all of these conditions.

### 3.2. PEG-Based Gel Modulus Controlled by H-Lys(alloc)-OH Concentration

PEGDA hydrogel mechanical properties were analyzed via static compression testing to evaluate the effects of including soluble H-Lys(alloc)-OH on the material modulus. For all gel conditions, PEGDA concentration was kept constant at 3.5% *w/v*, while H-Lys(alloc)-OH concentration was varied to achieve increasing molar ratios of allyl to acrylate. It was found that the addition of H-Lys(alloc)-OH reduced gel stiffness.

**Figure 2:**
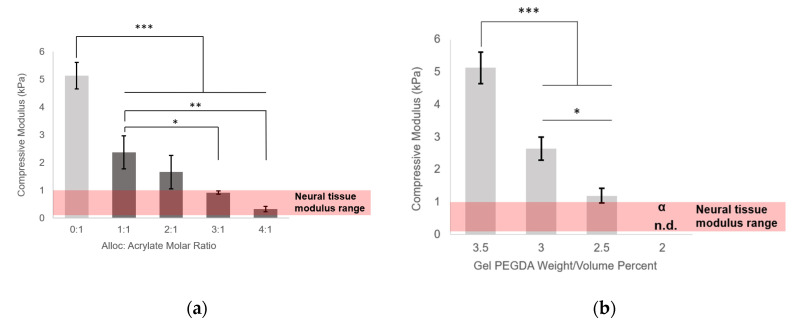
Hydrogel compressive moduli in response to H-Lys(alloc)-OH implementation and decreasing polymer density. Compressive modulus = slope of the stress–strain curve in the linear region following the toe region (Figure S1). Values reported as mean ± S.D. *n* = 4. * *p* < 0.05, ** *p* < 0.001, *** *p* < 0.0001, indicating a statistically significant difference. Statistical analysis evaluated by one-way ANOVA followed by Tukey–Kramer HSD tests. n.d. indicates no data, α: gels broke before evaluated strain region. (**a**) gel modulus reduced by increasing H-Lys(alloc)-OH concentration, neuronal stiffness achieved at highest monomer concentrations; (**b**) reducing polymer density decreases compressive modulus but does not reach neural tissue compliance.

The 3.5% *w*/*v* PEGDA control condition gels had an average modulus of 5.1 ± 0.48 kPa, which was decreased by adding soluble H-Lys(alloc)-OH to the gel precursor mixture (Figure 2a). With increasing alloc:acrylate, the gel compressive modulus was decreased in a concentration-dependent manner, with a compressive modulus of 0.92 ± 0.06 kPa at 3:1 alloc:acrylate (8.06 mg/mL H-Lys(alloc)-OH) and of 0.32 ± 0.09 kPa at 4:1 alloc:acrylate (10.75 mg/mL H-Lys(alloc)-OH), both within the range of neural tissue stiffness. The compressive modulus of 0.32 ± 0.09 kPa at 4:1 alloc:acrylate is a 93.7% reduction from the control condition.

In comparison, polymer density manipulation, a commonly used method of controlling polymer hydrogel mechanical properties, was implemented to determine the lowest modulus achievable by this method (Figure 2b). From 5.1 ± 0.48 kPa at 3.5% *w*/*v*, gel modulus was reduced in direct correlation to polymer density (2.6 ± 0.36 kPa at 3% and 1.2 ± 0.22 kPa at 2.5% *w*/*v*). Unlike the low stiffnesses achieved with H-Lys(alloc)-OH, 1.2 ± 0.22 kPa (exhibited by 2.5% gels) was the lowest compressive modulus achieved in PEGDA gels manipulated by decreasing polymer density. Below this polymer density, gels that formed were classified as unstable and broke before the evaluated strain region during testing, signified by α.

### 3.3. Influence on Modulus Is Dependent on Allyl-Containing Monomer Structure

**Figure 3:**
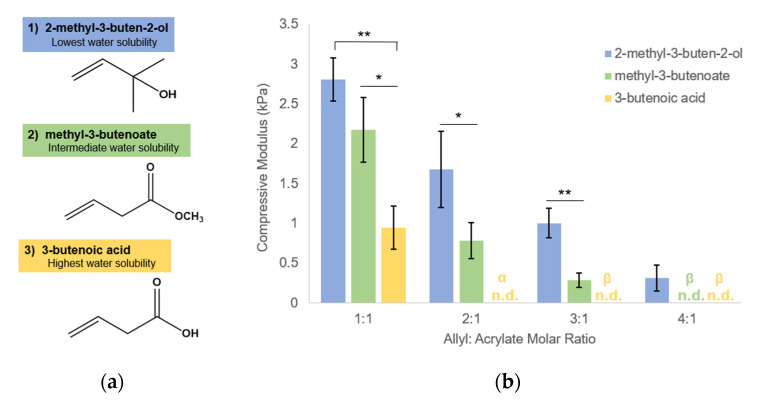
PEGDA modulus can be controlled by multiple allyl-containing monomers and increasing monomer polarity and monomer concentration both decrease hydrogel stiffness. (**a**) allyl-presenting monomer chemical structures; (**b**) compressive moduli of hydrogels. Compressive modulus = slope of the stress–strain curve in the linear region following the toe region. Values reported as mean ± S.D. *n* = 4. * *p* < 0.05, ** *p* < 0.001, indicating a statistically significant difference. statistical analysis evaluated by one-way ANOVA followed by Tukey–Kramer HSD tests. n.d. indicates no data, α: gels broke before evaluated strain region, β: gels did not form.

As Lys(alloc)-OH content controls gel mechanics via allyl-acrylate crosslinking competition, we tested whether this effect can be achieved with other allyl-presenting molecules, finding that this is possible with multiple monomers. Here, we demonstrated the effect on PEGDA hydrogel modulus of a range of concentrations of three different monomers possessing available allyl groups. Monomers tested were 2-methyl-3-buten-2-ol, methyl 3-butenoate, and 3-butenoic acid, which all contain allyl groups, but differ in structure and water solubility, as shown in Figure 3a. While gel stiffness decreased in response to increasing monomer concentration in a stepwise fashion for all three tested, competition efficiency and resultant effect on modulus were influenced by water solubility of the competitive monomer. For each concentration tested, 3-butenoic acid, which has the most polar of the 3 structures, caused the greatest reduction in gel modulus, followed by methyl 3-butenoate, then 2-methyl-3-buten-2-ol (Figure 3b). An average modulus of below 1 kPa was reached via implementation of each of the three monomers, with further modification to below 0.5 kPa possible with 2-methyl-3-buten-2-ol and methyl 3-butenoate.

### 3.4. Influence on Modulus Is Specific to Allyl-Presenting Monomers

**Figure 4:**
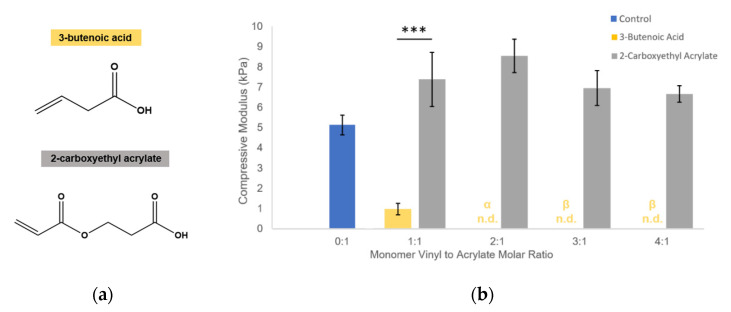
Allyl-presenting and acrylate-presenting monomers have significantly different effects on hydrogel modulus. (**a**) chemical structures of monomers; (**b**) compressive moduli of hydrogels with 3-butenoic acid and 2-carboxyethyl acrylate. Compressive modulus = slope of the stress–strain curve in the linear region following the toe region. Values reported as mean ± S.D. *n* = 4–6, *** *p* < 0.0001, indicating a statistically significant difference. Statistical analysis evaluated by Student’s *t*-test. n.d. indicates no data, α: gels broke before evaluated strain region, β: gels did not form.

To determine if the influence on gel stiffness was limited to allyl-presenting monomers, the effect of 3-butenoic acid (significant reduction in gel modulus, Figure 3b) was compared to that of 2-carboxyethyl acrylate, which has a matching structure to 3-butenoic acid except for the allyl group is replaced with an acrylate group (Figure 4a). It is known that acrylate groups have a higher tendency than allyls to propagate free radicals during polymerization because allyl groups have a greater likelihood to terminate the radical [30,39,40,41]; hence, this study served to investigate whether the competition was due to spatial/physical interference by any soluble monomers or due to the proposed mechanism of the monomer vinyl group influencing radical propagation (Figure 1d). In contrast to the effect of 3-butenoic acid, 2-carboxyethyl acrylate was shown to instead cause slight increases in modulus at all molar ratio conditions in comparison to the control condition (Figure 4b), suggesting that the competition mechanism is specific to allyl-presenting monomers.

### 3.5. Modulus Tuned Independently of Biomolecule Density

**Figure 5:**
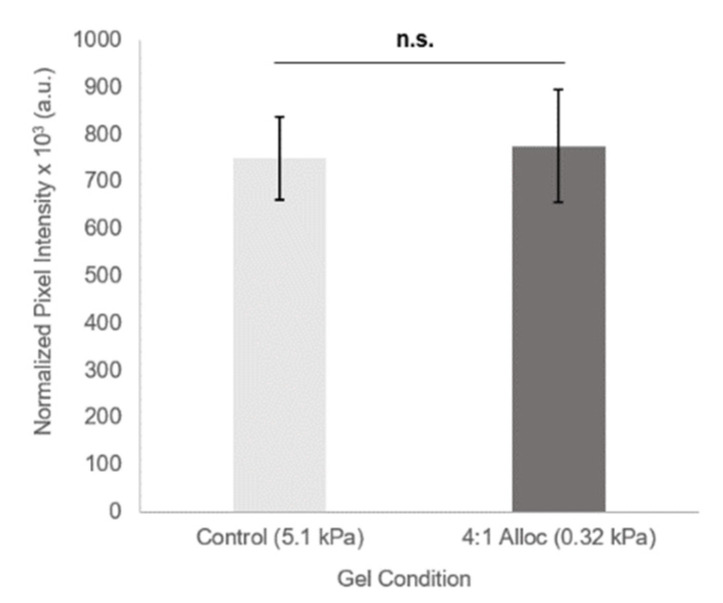
Biomolecule density with and without H-Lys(alloc)-OH competing monomer are comparable. Average fluorescence of gels at 488 nm, normalized to fluorescence of gels with 0 µM PEG-RGDS-Alexafluor 488. Values reported as mean ± S.D. n.s. indicates no significance. *p* = 0.8176, statistical analysis evaluated by Student’s *t*-test. *n* = 3 gels.

To determine that this system retains the decoupled nature of gel stiffness and biomolecule density (a key advantage of the PEG-based synthetic hydrogel system), we evaluated the effect of H-Lys(alloc)-OH inclusion on the quantity of PEG-RGDS incorporated into the gel network. Fluorescent PEG-RGDS-AlexaFluor 488 was included (7.5 µM) with PEGDA gel precursor with and without 10.75 mg/mL soluble H-Lys(alloc)-OH, corresponding to 4:1 alloc:acrylate. When gels were crosslinked for 50 sec, the average fluorescence intensity (normalized to fluorescence of PEGDA gels with 0 µM PEG-RGDS-488) did not significantly differ between the alloc (7.7 × 10^5^ ± 1.19 × 10^5^ a.u.) and control condition (7.5 × 10^5^ ± 0.88 × 10^5^ a.u.) (*p* value = 0.82), indicating the same RGDS concentration for both conditions (Figure 5).

### 3.6. H-Lys(alloc)-OH-Containing Materials Are Cytocompatible

**Figure 6:**
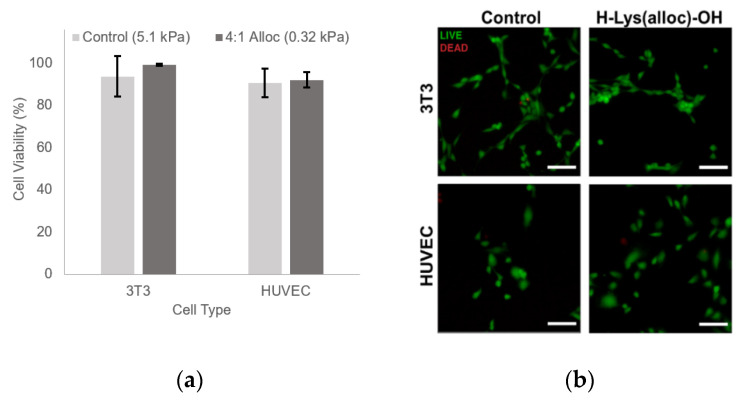
Addition of H-Lys(alloc)-OH does not significantly reduce cell viability. (**a**) percentage of live cells at 72 h after seeding. Values reported as mean ± S.D. *n* = 4. n.s. indicates no significant difference. 3T3: *p* = 0.35, HUVEC: *p* = 0.74. Statistical analysis evaluated by Student’s *t*-test; (**b**) Live/Dead images at 72 h after seeding. Scale bar = 100 µm.

Upon adding a new monomer component to the hydrogel system, material cytocompatiblity was assessed. NIH 3T3 fibroblasts seeded on 0.32 ± 0.09 Pa gels fabricated with 4:1 alloc:acrylate (10.75 mg/mL H-Lys(alloc)-OH) exhibited 99.2 ± 0.42% 72 h after seeding, as determined from quantification of Live/Dead staining (Figure 6a). This did not differ significantly from the 93.65 ± 9.57% average viability of 3T3s seeded on the PEGDA control gels at the same timepoint (*p*-value = 0.35). HUVECS seeded on gels of the same conditions demonstrated similar high viability across both conditions, with 90.5 ± 6.77% viability on control gels and 92.09 ± 3.65% viability on alloc gels (*p*-value = 0.74). Figure 6b shows representative Live/Dead staining images of each condition.

### 3.7. Allyl-Controlled Gel Stiffness Influences Cell Behavior and Morphology

#### 3.7.1. Fibroblast Spreading Reduced with Decreasing Gel Stiffness, Further Decreased on Softest Alloc Gels

**Figure 7:**
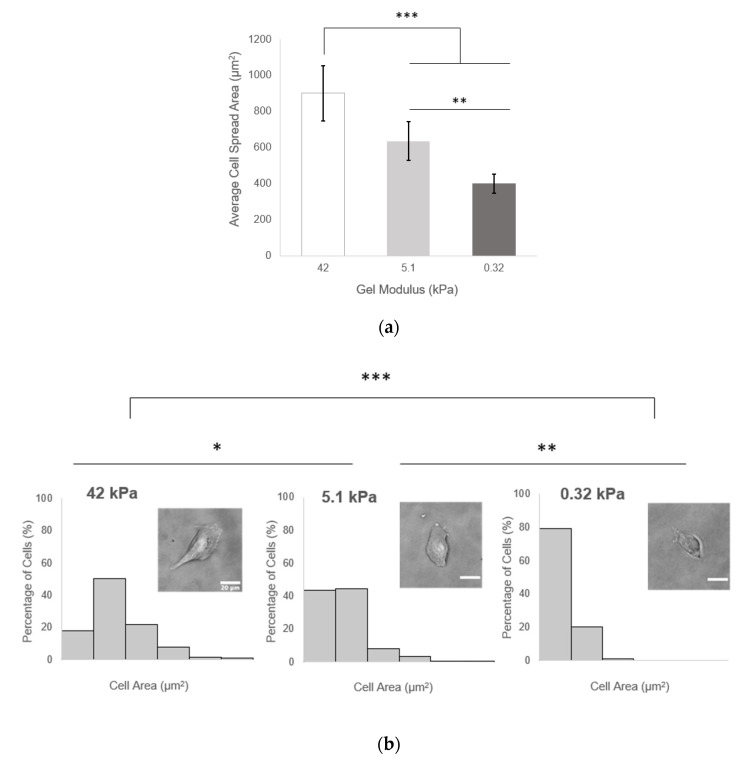
Fibroblast spreading is directly related to hydrogel stiffness. (**a**) average cell area at 24 h after seeding. Values reported as mean ± S.D. ** *p* < 0.001, *** *p* < 0.0001, indicating a statistically significant difference. Statistical analysis evaluated by one-way ANOVA followed by Tukey–Kramer HSD tests. *n* = 11 gels, >18 cells counted per gel; (**b**) distributions of cell spread areas 24 h after seeding. * *p* < 0.0167, ** *p* < 0.001, *** *p* < 0.0001, indicating a statistically significant difference between all distributions as evaluated by pairwise Kolmogorov–Smirnov asymptotic tests with Bonferroni correction. Average cell area at 24 h after seeding. Insets: representative images of cell for each condition. Scale bar = 20 µm.

Fibroblasts are known to exhibit greater spreading and migration on stiffer substrates and reduced spreading in soft tissue-like environments [3]. Here, alloc-acrylate competition was implemented to increase PEGDA-PEG-RGDS gel compliance, which resulted in decreased fibroblast spreading. The 3T3 fibroblasts exhibited the highest displayed average spread area per cell of 901.72 ± 153.19 µm^2^ after 24 h when seeded on 10% *w*/*v* PEGDA gels with a compressive modulus of 42 ± 3.17 kPa—within the range of immature bone tissue stiffness [42]. Cells seeded on control gels at 5.1 ± 0.48 kPa showed a significantly lower average spread area of 637.02 ± 108.27 µm^2^. Cells seeded on 0.32 ± 0.09 Pa gels with 4:1 alloc demonstrated an average cell spread area of 401.28 ± 53.33 µm^2^, a 37% reduction in average spread area from the control condition and a 55.5% reduction from the 10% PEGDA condition (Figure 7a). The binned distributions of cell spread areas, shown in Figure 7b along with representative phase images of cells seeded on each gel condition, were found to be significantly different from one another by pairwise Kolmogorov–Smirnov tests.

#### 3.7.2. Most Compliant H-Lys(alloc)-OH Gels Support Neurite Outgrowth

**Figure 8:**
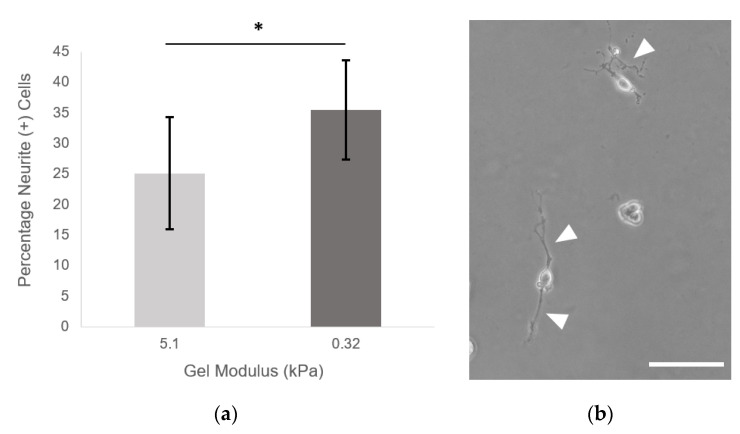
Neurite outgrowth is increased on softest hydrogels. (**a**) PC-12 neurite outgrowth as a function of gel modulus. Neurite (+) cell = has at least one neurite ≥ 2× the cell body diameter. Data shown as mean ± S.D. * *p* < 0.05, indicating a statistically significant difference. Statistical analysis evaluated by Student’s *t*-test, *n* = 9 gels; (**b**) PC-12 cells on 0.32 ± 0.09 kPa gels at day 7. Arrowheads indicate neurites. Scale bar = 100 µm.

Neural cells favor neurogenesis and neurite extension on very soft substrates, below 1 kPa modulus [11,13,14,15,16]. In this study, PC-12 neuronal cells seeded on PEGDA-PEG-RGDS gels demonstrated significantly increased neurite extension in response to decreased gel modulus. An average of 25.14 ± 9.20% of cells per gel cultured on 5.1 ± 0.48 kPa control gels extended neurites greater than 2× the length of cell body at 7 days after seeding. Gels incorporating alloc to present a 0.32 ± 0.09 kPa substrate to PC-12 cells resulted in 35.51 ± 8.18% of cells extending neurites at Day 7, a 41% increase from the control condition (Figure 8a). Figure 8b shows representative neurites on 0.32 ± 0.09 kPa hydrogels; white arrowheads indicate neurites.

## 4. Discussion

Creating cell culture platforms for investigation of soft tissue cell behavior, such as neural cell behavior, in response to environmental cues will significantly contribute to progress in developing tissue engineered constructs for directing therapeutic regeneration. To most accurately model the natural soft tissue environment, cell culture matrices must match the mechanical properties of in vivo soft tissue. Current systems for neural cell culture that match the mechanical stiffness of the brain are mainly limited to naturally-derived polymer matrices such as collagen and fibrin gels [25,26,27,28,29]. These types of materials present a myriad of biological signals that vary between batches and cannot be uncoupled from polymer density (and thus mechanical properties), which can confound findings. To fully parse apart the influence of all environmental signals, both individually and in concert, the ideal cell culture matrix is not just highly compliant, but also a reproducible system with independent control over biological and mechanical properties.

Synthetic PEG-based hydrogels, while providing greater control over each material property, are less mechanically appropriate for neural cell culture because they generally cannot form crosslinked gels at low enough stiffnesses to accurately model soft tissue [30]. The objective of this study was to expand customizable synthetic PEG-based hydrogel platforms to soft tissue culture applications by implementing a method of independently and tightly controlling material stiffness to within the soft tissue range without influencing biochemical signaling.

Here, we demonstrated the ability to control PEG-based hydrogel stiffness down to 0.32 ± 0.09 kPa compressive modulus by including H-Lys(alloc)-OH, a small, allyl-based monomer, in the hydrogel precursor solution. In PEGDA gelation, the acrylate endgroups polymerize to form crosslinked poly(acrylic acid) nodes connected by PEG backbones (Figure 1b). While allyl-based monomers can participate in the addition reactions, they have a greater propensity to terminate rather than propagate the free radical reaction as compared to acrylate-based monomers, altering the connectivity of the network of crosslinking nodes [30,39,40,41]. The concentration of monomers (and thus, soluble allyl to PEGDA acrylate molar ratio) was altered to finely control the stiffness of soft PEGDA-based gels even within the modulus range of neural tissue (0.92 ± 0.06 kPa at 3:1 alloc:acrylate and 0.32 ± 0.09 kPa at 4:1 alloc:acrylate) (Figure 2a). This precise control, previously unachievable by conventional methods of PEGDA hydrogel stiffness manipulation, is particularly advantageous for investigating neural cell differentiation. Differences in tissue stiffness within the soft tissue regime can lead to diverging differentiation between astrogenesis and neurogenesis [13], and for therapeutic applications, a controlled ratio of neuron to astrocyte production is optimal for functional recovery.

We propose that the soluble allyl monomers compete with the acrylate groups to participate in crosslinking and cause early termination at crosslinking nodes, altering the hydrogel crosslinking mechanics. Our findings that incorporation of multiple different allyl-presenting monomers reduce PEGDA hydrogel stiffness (Figure 3), while including an acrylate-presenting monomer (with an otherwise matching monomer structure) increases hydrogel modulus (Figure 4) and supports this proposed mechanism, indicating that the reductions in gel stiffness are caused by allyl-specific polymerization competition.

We have previously shown that allyl-acrylate competition can be used to reduce PEGDA hydrogel stiffness, where the allyl group was presented as a side group on the PEG backbone [30]. By transitioning to soluble presentation of competitive allyl groups, our current study provides a facile approach to exert more precise control over hydrogel modulus within the stiffness regime of natural soft tissue. We show that the concentration of the allyl monomer can be adjusted to achieve different stiffnesses, and that different hydrophilic allyl monomers can be switched in to adjust stiffness as well based on their structure (Figure 4). The control and customization expand the application potential for this technology in the field of soft tissue engineering.

Because it does not control mechanical stiffness by altering polymer density, this method softens the hydrogels without preventing network formation or influencing other material properties like diffusivity or immobilization of bioactive factors. In addition to forming self-supporting soft gels, this system retains independent tunability of stiffness and biochemical presentation. The concentration of fluorescent PEG-RGDS incorporated into the gel did not differ significantly between gels with and without alloc (Figure 5). This allows us to explore the effect of mechanical properties via allyl-acrylate competition independent of biomolecule density.

Beyond showing that hydrogels containing Lys(alloc)-OH are cytocompatible and suitable for use as cell culture platforms, we demonstrated that cells seeded on these compliant matrices demonstrated behavior representative of cells in soft-tissue like environments. Here, fibroblast cells displayed more rounded morphology on the softest hydrogels (highest alloc concentration) than those on stiffer PEGDA hydrogels, consistent with previous studies of cell spreading on substrates modeling soft and stiff tissue environments [3]. By causing a stiffness-mediated decrease in fibroblast spreading, this system demonstrates that its potential as an appropriate model of soft tissue environments. Furthermore, in many studies of neural cell and neural stem/progenitor cell behavior, neuronal differentiation and neurite outgrowth has been consistently shown to be increased on softer substrates and optimized at stiffnesses matching that of in vivo neural tissue, in the range of 0.1–1 kPa [11,13,14,15,16]. Here, we saw a significant increase in the number of neurite (+) PC-12 cells when the cells were seeded on the softest gels at 0.32 ± 0.09 kPa, indicating that, via addition of alloc into the gel mixture, we are able to create a system that can support neural cell culture and neurite outgrowth at stiffnesses previously not possible with PEG-based gels.

## 5. Conclusions

In this work, we demonstrated a method for reducing synthetic PEGDA hydrogel stiffness to that of natural neural tissue while maintaining stable gel formation and independent control over material properties. Incorporating soluble, allyl-presenting monomers in the hydrogel precursor solution results in allyl-acrylate competition that alters crosslinking mechanics to decrease hydrogel bulk stiffness. In addition, precise control over modulus can be achieved by modulating the monomer concentration and using monomers with differing polarity. Gels made with H-Lys(alloc)-OH as the competitive monomer were shown to be cytocompatible and to support cell culture. When hydrogel modulus was tuned to 0.32 ± 0.09 kPa compressive modulus, fibroblasts cultured on the hydrogels demonstrated a decrease in cell spreading and neural PC-12s displayed an increase in neurite extension. These soft tissue-representative cell behaviors demonstrate the potential for this compliant hydrogel system to be used as a platform for investigating soft tissue cell behavior, and specifically neural cell behavior, in a highly controlled environment towards developing regenerative tissue engineered constructs for soft tissue regeneration.

## Supplementary Materials

The following are available online at https://www.mdpi.com/2073-4360/12/12/3000/s1. Figure S1: Stress-strain curves for PEGDA hydrogels.
