# Supplementary material for: Modulating Functionalized Poly(ethylene glycol) Diacrylate Hydrogel Mechanical Properties through Competitive Crosslinking Mechanics for Soft Tissue Applications
Source: Polymers (Basel). 2020 Dec 16;12(12):3000. doi: 10.3390/polym12123000 (PMC7766244; doi:10.3390/polym12123000)
Supplement: Supplementary file 1 [file polymers-12-03000-s001.pdf]

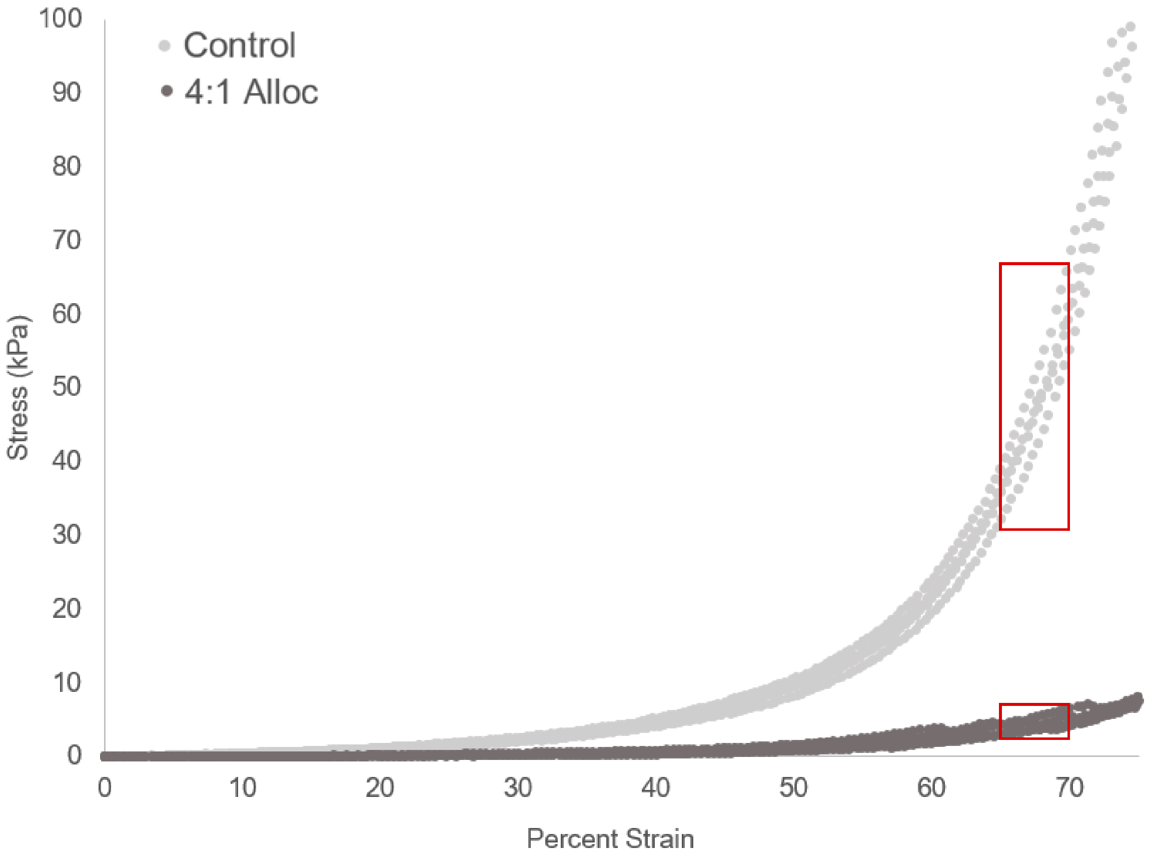

Figure S1. Stress-strain curves for PEGDA hydrogels. 0:1 (control) and 4:1 alloc to acrylate conditions. It can be seen that the hydrogels undergoing compression experience an elongated toe region before reaching the linear region. This linear region, corresponding to 65-70% strain, is outlined with a red box. The slope of this linear region was taken as the compressive modulus of each hydrogel.
